# First complete mitochondrial genomes of molossid bats (Chiroptera: Molossidae)

**DOI:** 10.1080/23802359.2017.1298419

**Published:** 2017-03-10

**Authors:** Vanessa A. Mata, Francisco Amorim, Antonio Guillén-Servent, Pedro Beja, Hugo Rebelo

**Affiliations:** a CIBIO-InBIO, Centro de Investigação em Biodiversidade e Recursos Genéticos, Universidade do Porto, Vairão, Portugal;; b Departamento de Biologia, Faculdade de Ciências, Universidade do Porto, Porto, Portugal;; c Instituto de Ecología, Xalapa, México;; d CEABN-InBIO, Centro de Ecologia Aplicada ‘Professor Baeta Neves’, Instituto Superior de Agronomia, Universidade de Lisboa, Lisboa, Portugal;; e School of Biological Sciences, University of Bristol, Bristol, UK

**Keywords:** Molossidae, mitogenome, *Tadarida teniotis*, *Tadarida latouchei*, *Chaerephon plicatus*

## Abstract

Bats represent around one-fourth of the world’s mammals and their taxonomy is still controversial. Molossids are one of the most diverse bat families with a wide knowledge gap. In this study, we report the first complete mitochondrial genomes of three molossid bats: the European free-tailed bat *Tadarida teniotis*, the La Touche’s free-tailed bat *Tadarida latouchei*, and the Wrinkle-lipped free-tailed bat *Chaerephon plicatus*. The mitogenomes are 16,869 and 16,784 bp long for *T. teniotis* and *T. latouchei*, respectively, while in *C. plicatus* it is at least 16,216 bp although the control region was not fully recovered due to its higher divergence from *T. teniotis*. The genomes show conserved synteny with other mammalian mitogenomes, containing 13 protein-coding genes, 2 ribosomal RNA genes, 22 transfer RNA genes, and 1 control region (d-loop). All protein-coding genes start with the ATG start codon, except for ND2, ND3, and ND5 which begin with ATA or ATT. Eleven protein-coding genes terminated in a canonical stop codon, TAA or TAG, two contain incomplete stop codons, T or TA. Cytochrome b terminates in the mitochondria-specific stop codon AGA. These mitogenomes provide a valuable resource for future studies of Molossidae and other bat and mammal species.

Molossidae is the fourth largest bat family (Mammalia, Chiroptera), with ∼100 species divided into 17 genera (IUCN 2017). Although molossids are distributed throughout tropical and subtropical regions of the world, the range of most species is poorly known. Many species are similar in appearance and difficult to capture due to their fast and high-flying behaviours (Vaughan 1966). This has led to an under-representation of this family in museum collections and several taxonomic inconsistencies (Ammerman et al. 2012). In the Eurasian region there are at least three recognized species of free-tailed bats: *Tadarida teniotis* complex (composed of *Tadarida teniotis*, *Tadarida insignis*, *Tadarida latouchei,* and *Tadarida coecata*), *Tadarida aegyptiaca* and *Chaerephon plicatus* (Hutson et al. 2001). The *teniotis* species group has been subject to great debate, with some authors considering *insignis, latouchei* and *coecata* as subspecies of *teniotis*, while more recently others consider *insignis* and *latouchei* as full species, and *coecata* as a subspecies of *insignis* (Simmons 2005). Morphological analysis of *teniotis*, *insignis*, and *latouchei*, have found consistent differences, suggesting the full species status for each group, and therefore the restriction of *teniotis* to the west of India, and *insignis* to further east (Funakoshi & Kunisaki 2000). However, to date this classification has lacked any molecular support. In fact, there are no available mitogenomes for any species of the family Molossidae. Our results provide useful reference for future phylogenetic and phylogeographic studies.

Genomic DNA was extracted from *T. teniotis* (Portugal, 41.287 N 6.873 W), *T. latouchei* (Laos, 20.153 N 103.407 E, voucher ROM MAM 118321), and *C. plicatus* (Laos, 20.723 N 101.138 E, voucher ROM MAM 118373) tissue samples using QIAamp DNA *Micro Kit* (QIAGEN). Due to the degraded state of *T. latouchei* and *C. plicatus* samples, a mitochondrial capture-based protocol was used. Specific primers for *T. teniotis* were designed in order to amplify 2 overlapping fragments of ∼10kb (MtF13 (5′-TGCATTACACATCCGACACA-3′) with MtR12 (5′-GGCTTTGAAGGTCCTTGGTC-3′), and MtF12 (5′-CGGCTAACATACGCTACATCC-3′) with MtR13 (5′-GCCTATGAAGGCAGTGGCTA-3′) using Takara LA *Taq* polymerase. Custom primers had to be designed due to several problems in the amplification process related to nuclear copies of mitochondrial genes. Complete mitochondrial mitogenomes of each sample were then captured following the protocol by Maricic et al. (2010) and sequenced with 250 bp paired end reads in Illumina MiSeq (Vairão, Portugal). Due to the high divergence between *T. teniotis* and *C. plicatus*, the control region d-loop of C. *plicatus* mitochondria was not fully recovered. The mitogenomes (Genbank accession no. KY581660/61/62) were assembled *de novo* and annotated using Geneious 9.1.5 (Kearse et al. 2012). A neighbour-joining tree was built with the Tamura–Nei distance to reconstruct the phylogeny of the molossidae family using Geneious based on an alignment of 55 bat species mitogenomes (without d-loop) plus the ones from this study (Saitou & Nei 1987).

Molossidae were more closely related to Vespertilionidae (Figure 1), with *Tadarida* species forming a separate clade from *Chaerephon*. Average coding gene distance between *T. teniotis* and *T. latouchei* and *C. plicatus* was 10% and 15%, respectively. These results suggest that *T. latouchei* is indeed a different species from *T. teniotis*, although nuclear markers would be needed to confirm this. Molossidae are one of the most diverse family of bats, however these are the first mitogenomes available. Future studies with more taxa and molecular markers are needed to better understand the phylogeny and the taxonomic status of this highly speciose family of mammals.

**Figure 1.**
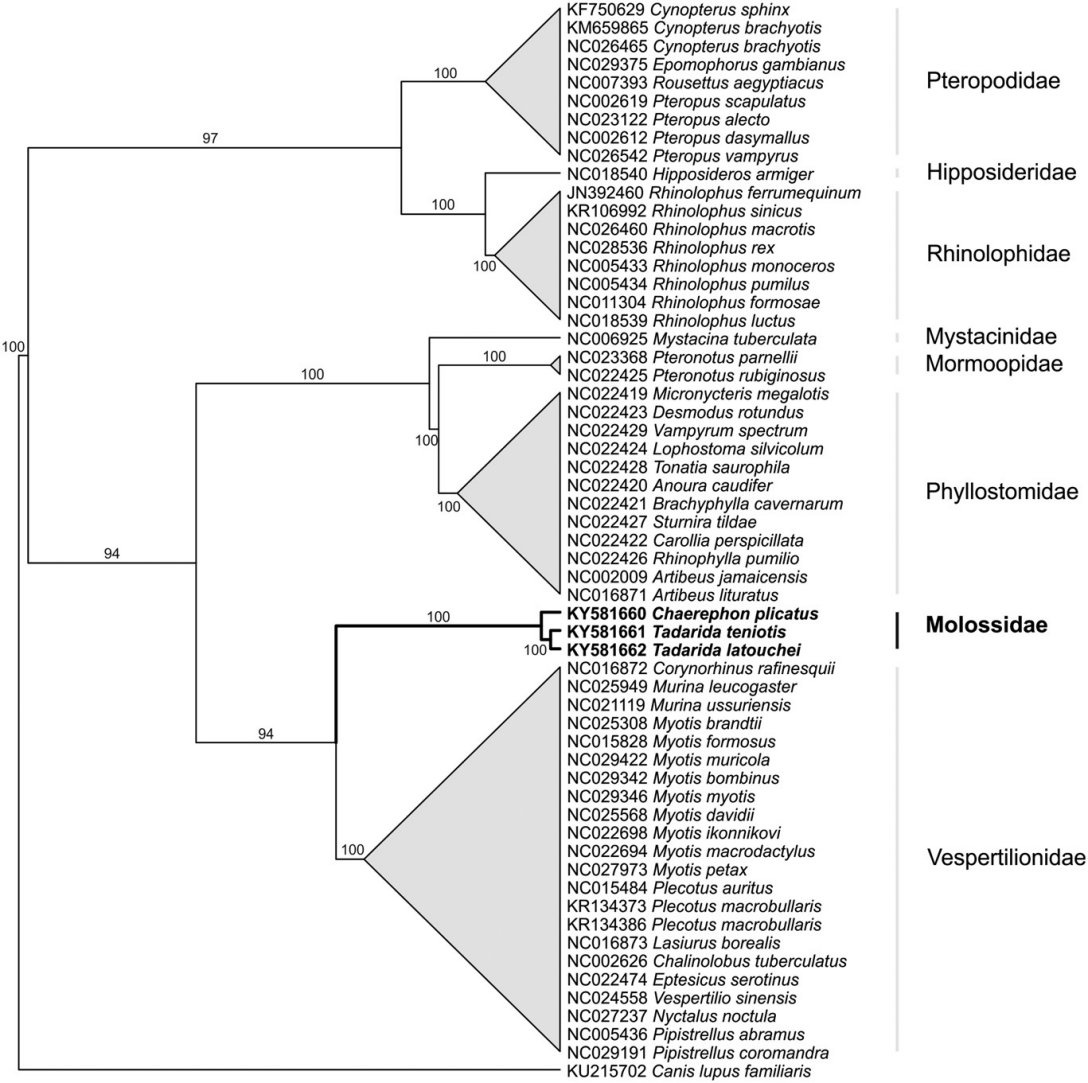
Phylogenetic relationship between Molossidae and other bat species. Phylogenetic tree constructed using neighbour-joining method with 58 bat mitogenomes. All species cluster into their expected taxonomic family. Numbers at branches indicate bootstrap support.
